# A Copper-Molybdenum Etchant with Wide Process Window, Long Bath Life and High Stability for Thin Film Transistor Liquid Crystal Display Applications

**DOI:** 10.3390/ma18081795

**Published:** 2025-04-14

**Authors:** Bing Zhang, Yafen Yang, David Wei Zhang

**Affiliations:** 1 School of Microelectronics, Fudan University, Shanghai 200433, China; 18112020056@fudan.edu.cn; 2 Jiashan Fudan Institute, Jiaxing 314100, China

**Keywords:** copper-molybdenum etchant, TFT-LCD manufacturing, process window optimization, copper ion loading, bath-life enhancement

## Abstract

Conventional etchants for multi-metal/alloy stacked structures often suffer from nonuniform etching, residual layers, or undercutting, failing to meet high-generation production standards. This study presents a stable copper-molybdenum (Cu-Mo) etchant with extended bath life for thin film transistor liquid crystal display (TFT-LCD) applications, achieved through compositional optimization. Systematic investigations have been conducted on the effects of etching time, copper ion (Cu^2+^) loading (bath life) and storage time on the etch performance, alongside evaluations of sudden-eruption point and material compatibility. Results demonstrate that over-etching beyond the “detected endpoint” by 10% to 90% maintains critical dimension (CD) bias and taper angle of MoNiTi(MTD)/Cu/MTD three-layer and Cu/MTD two-layer within process specifications, as well as the difference between the CD bias of the three-layer and two-layer structures at the same over-etch time. The optimized formulation exhibits a 20% broader process window and 20% longer bath life compared to the process-of-record (POR) etchant. Shelf stability exceeds 15 days with minimal performance degradation, while maintaining compatibility with industrial equipment materials. These advancements address key challenges in high-precision etching for advanced TFT-LCD manufacturing, providing a scalable solution for next-generation display production.

## 1. Introduction

Recent advancements in thin film transistor liquid crystal display (TFT-LCD) technology have driven the demand for panels with higher resolution, enhanced image quality, and reduced energy consumption. To achieve these goals, manufacturing processes must produce devices with finer pixel densities and faster signal response times [1,2]. This necessitates electrode materials with exceptional electron transfer efficiency and stability. Copper (Cu), owing to its low resistivity (about 40% lower than aluminum (Al)) and high electron mobility, has emerged as the preferred replacement for Al in TFT metal electrodes [3]. However, Cu’s poor adhesion to glass substrates and tendency to diffuse into underlying layers under thermal stress have necessitated the use of barrier materials such as molybdenum (Mo), titanium (Ti), or their alloys. These materials are interposed between Cu and the glass substrate, forming robust stacked structures that prevent interfacial delamination and Cu migration [4,5,6]. The resulting Cu/Mo-based multilayers combine Cu’s superior thermal conductivity with Mo’s low thermal expansion coefficient, enabling precise control over the thermal expansion, thermal conductivity and heat dissipation through layer thickness optimization [7]. Such structures are now integral to gate and source/drain metallization in high-performance TFT arrays, where tailored resistance and response speeds are critical for applications ranging from displays to automotive panels.

Despite their advantages, Cu/Mo stacked structures present significant etching challenges in high-generation production lines. Wet etching using weakly acidic hydrogen peroxide (H_2_O_2_)-based etchant—commonly employed in back-channel etch processes—faces escalating demands as panel sizes increase and critical dimensions (CDs) shrink [8]. The reduced margin for error in TFT array fabrication narrows the allowable process window, requiring unprecedented control over etching uniformity across vast panel areas. Simultaneous etching of heterogeneous material interfaces (Cu/Mo, Cu/Ti) and complex reaction kinetics at different etching stages often lead to nonuniform material removal. This manifests as metal residues, undercutting, excessive CD bias, or irregular taper angles, all of which violate stringent process specifications [9,10,11,12,13,14,15,16]. Metal residues can affect the morphology of the passivation layer in subsequent process, and even cause passivation layer fracture, affecting product quality. Compounding these issues, rising copper ion (Cu^2+^) concentrations in the etchant bath—due to cumulative substrate processing—destabilize etching performance. For instance, Cu^2+^ loading beyond 5000 ppm accelerates taper angle drift and CD variation, particularly when etching thick Cu films (>5500 Å). Suboptimal taper angles compromise step coverage during subsequent passivation layer deposition, increasing the risk of microcracks or line breaks. Similarly, CD deviations directly impair device functionality, linewidth nonuniformity alters TFT threshold voltages, while interlayer CD mismatches induce signal transmission delays, resulting in visible artifacts such as Mura [17,18]. These challenges underline the urgent need for etchants that simultaneously achieve broad process windows, high Cu^2+^ loading (long bath life), high stability and compatibility with diverse metal stacks—a requirement unmet by conventional formulations.

Building on prior investigations into component-specific etching mechanisms, this study presents a systematically optimized copper-molybdenum (Cu-Mo) etchant designed to address the limitations of existing solutions. Through combinatorial adjustment of etchant compositions—including H_2_O_2_ stabilizers, metal-specific chelators, and pH regulators—we developed formulations that enable precise control over etch rates, taper angles, undercutting and CD bias. Key innovations include the integration of organic stabilizers to mitigate Cu^2+^-induced autocatalytic decomposition, extending bath life by 20% compared to process-of-record (POR) etchants. Additionally, optimized chelator concentrations suppress undercutting and residue formation by modulating surface passivation kinetics. Experimental validation confirms that the etchant maintains CD bias and taper angles within process specifications even under extended over-etch percentage (OE) conditions (10–90% beyond endpoint detection). Furthermore, the formulation demonstrates robust compatibility with G4.5–G8.5 production lines. Stability testing reveals a shelf life exceeding 15 days without performance degradation, ensuring compatibility with industrial storage. By resolving the trade-offs between etch selectivity, process window breadth, and bath stability, this work establishes a foundation for scalable high-precision etching in next-generation displays, particularly those targeting 8 K resolution and 120 Hz refresh rates.

## 2. Materials and Methods

The test samples used in this study comprised diverse substrate panels provided by panel manufacturers with films deposited through magnetron sputtering and patterning, including two-layer structures such as Cu/Mo, Cu/MoNiTi, Cu/MoTi, Cu/MoNb, as well as three-layer structures such as Mo/Cu/Mo, MoNiTi(MTD)/Cu/MoNiTi, MoTi/Cu/MoTi. The Cu layer thickness ranges from 3000 Å to 8000 Å. The bottom layer thickness of Mo, MoNiTi or MoTi alloy ranges from 200 Å to 400 Å, while the top layer thickness ranges from 100 Å to 300 Å. The test substrate panels come from G4.5, G6, and beyond generations such as M1 and M2 processes of G8.5 generation lines. To streamline manufacturing and reduce costs, panel manufacturers now employ a four-mask process, wherein the source/drain metal lines and amorphous silicon semiconductor layer share a common masking step. This necessitates two sequential wet etching steps: the M2 first wet etch (data lines and channel outer etching) and M2 second wet etch (channel inner etching) [19]. The requirement to etch these substrate panels separately exacerbates CD loss in source/drain metal lines and increases susceptibility to undercutting. Given the extensive diversity of glass substrate panels in this study, developing a universal Cu-Mo etchant capable of addressing all etching performance requirements presents significant challenges. The etchant must simultaneously ensure compatibility with heterogeneous material systems and mitigate CD loss across multiple etching stages.

In this study, test samples are cut into dimensions ranging from 3 cm × 4 cm to 7 cm × 7 cm for laboratory setups. Etchants are prepared by dissolving the raw materials of various components such as hydrogen peroxide, fluoride, azole, organic acids, inorganic salts, alcohol ether, amine, etc., which are analytical grade or electronic grade reagents, in deionized water according to designed formulations. POR etchant used in panel manufacturing is a proprietary product with confidential information, and difficult to obtain. This study uses POR etchant as a benchmark to compare the etchants we have developed. Prior to etching test, the proposed etchant is preconditioned by doping with Cu^2+^ and Mo^3+^ ions to predetermined concentrations, achieved either by etching Cu/Mo dummy panels or dissolving pure metal powders. The Cu^2+^/Mo^3+^ ratio is typically maintained at 20:1 to stabilize etching kinetics—a critical step for mitigating rate fluctuations and ensuring process repeatability. Industry empirical data indicate that 300–500 ppm is the optimal Cu^2+^ preconditioning levels to stabilize etching kinetics, mitigate rate fluctuations and ensure process repeatability, accordingly, this study utilized 300 ppm or 500 ppm Cu^2+^ concentrations as baseline conditions. Etching processes are conducted at tightly controlled temperatures of 30–32 °C. Etch duration is defined relative to the endpoint detection time (EPD), corresponding to the moment when optical inspection confirmed complete metal layer removal. OE intervals—expressed as percentages beyond EPD. Post-etch, samples underwent sequential rinsing with deionized water and N_2_ drying to prevent oxidation artifacts before characterization.

**Figure 1:**
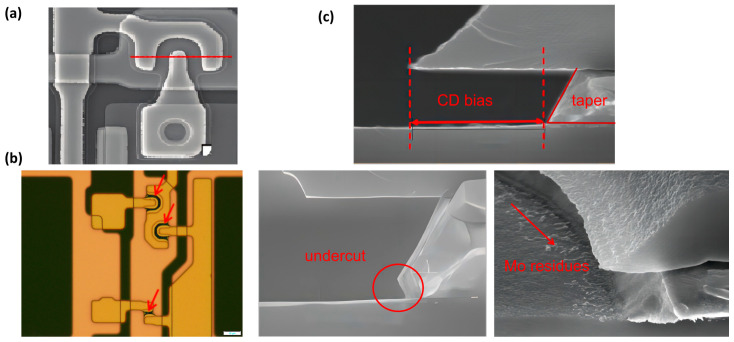
(**a**) Top view of the sample, with dashed lines indicating the cutting positions for obtaining a cross-sectional SEM. (**b**) Comparison of the M2 second wet etch area with the M2 first wet etch area, highlighting the black channel position indicated by the arrow. (**c**) Schematic diagrams of taper angle, CD bias, undercut, and Mo residues.

Scanning electron microscopy (SEM, Hitachi S-4800) is mainly used to characterize the etching results of metal lines. When preparing SEM samples, the test pieces are cut perpendicular to the direction of the metal lines, as shown in Figure 1a. SEM observations are conducted in either a cross-sectional view or a top view tilted at 30°. As shown in Figure 1b, the M2 second wet etch area is compared to the M2 first wet etch area, with the black channel position indicated by the arrow. As shown in Figure 1c, cross-sectional SEM images are used to measure the taper angle of etched metal line profile, the distance from the edge of the photoresist on the top of metal line to the bottom edge of the metal line (CD bias), and to observe the straightness of the etched profile, including undercut and tails. In top-view SEM images, the primary focus is to check for the existence of metal residues below the photoresist or in the channel areas. When necessary, an X-ray energy dispersive spectrometer (EDS, Thermo Scientific Helios G4 CX) can be employed to further investigate the tail section of the etched metal lines and the channel area, identifying both the presence and type of metal residues. Multiple locations on each sample are observed to ensure the representativeness of SEM and EDS results. And each data point of CD bias and taper angles presented in this paper is the average of 6 data points, and the standard deviation is calculated.

## 3. Mechanism Analysis

### 3.1. Mechanism of H_2_O_2_ Decomposition Catalyzed by Cu^2+^

**Figure 2:**
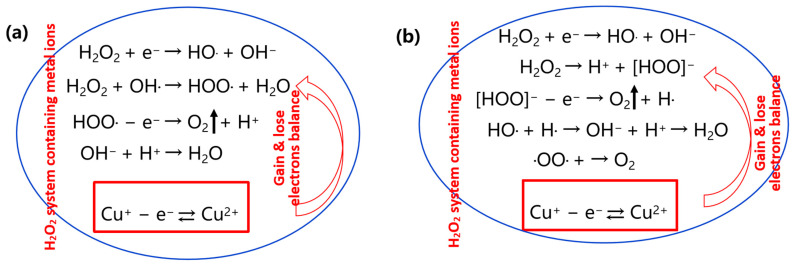
Schematic diagram of Cu^2+^ catalyzing the decomposition of H_2_O_2_. (**a**) Structural self-decomposition (activation reaction); (**b**) ionizing decomposition (free radical reaction).

The interactions between etchant components are inherently complex, requiring systematic analysis to understand their collective impact on etching performance. H_2_O_2_ is a strong oxidant and reacts with metals such as Cu, Mo, Ti, and their alloys, generating corresponding metal oxides. Inorganic or organic acids facilitate the etching process by providing hydrogen ions (H^+^) and establishing an acidic environment. These conditions enable the chemical reaction of the acids with metallic substrates and their oxides, converting insoluble metals and metal oxides into soluble metal salts, achieving the purpose of etching metals. As the number of processed glass panels increases during etching, the concentration of metal ions, particularly Cu^2+^, in the etchant rises proportionally. This accumulated Cu^2+^ acts as a persistent catalyst for the self-decomposition of H_2_O_2_, significantly accelerating its breakdown and introducing risks of exothermic reactions or even explosive conditions. The catalytic mechanism of Cu^2+^ in H_2_O_2_ decomposition operates through two primary pathways: activation reactions and free-radical reactions. In activation reactions, Cu^2+^ coordinates with H_2_O_2_ molecules, weakening the O–O bond and facilitating cleavage, thereby accelerating structural self-decomposition. Concurrently, free-radical reactions involve the generation of hydroxyl radicals (·OH) and superoxide radicals (·O_2_^−^), which further destabilizes H_2_O_2_ by ionizing decomposition [20,21]. A detailed schematic of this electron-transfer-mediated reaction pathway is illustrated in Figure 2.

### 3.2. Stabilizing Effect of H_2_O_2_ Stabilizers and Metal Chelators

Therefore, H_2_O_2_ stabilizers and metal chelators are added to the etchant to suppress the decrease in Cu etch rate caused by H_2_O_2_ decomposition, the decrease in the number of processed glass panels, and the instability arising from exothermic reaction of H_2_O_2_ in the case of increasing Cu^2+^ concentration. It is found that alcohol ether compounds, compared to other substances, such as organic phosphonic acids, ureas, inorganic salts, and lower alcohols, exhibit superior stabilization effects on H_2_O_2_ [22,23,24,25]. These compounds do not introduce poorly soluble components or metal ions that may affect Cu lines, nor do they react with other key components in a way that would compromise their functionality. During etching experiments, it is observed that in the proposed formulations the etchant without addition of alcohol ether compounds rapidly released a large number of large bubbles, while the etchant containing alcohol ether compounds produced fewer, smaller bubbles at a slower rate. Moreover, when alcohol ether compounds like triethylene glycol are used as H_2_O_2_ stabilizer in the proposed formulations, variations in their concentration have minimal impact on key etching characteristics, such as etch rate, CD bias, and taper angle, provided their concentration remains within an appropriate range.

**Figure 3:**
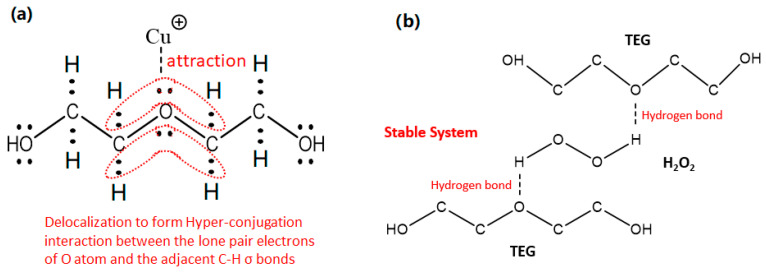
Theoretical explanation for the stabilization of H_2_O_2_ by alcohol ether compounds: (**a**) complexation between TEG and Cu^2+^; (**b**) the stabilizing effect of hydrogen bonds between TEG and H_2_O_2_.

Alcohol ether compounds, such as triethylene glycol (TEG) and diethylene glycol, have both alcohol and ether properties due to the presence of ether and hydroxyl groups. These compounds can stabilize excess Cu^2+^, preventing reaction with H_2_O_2_ and improving the stability of the etch process. As shown in Figure 3a, the C-H σ-bond of alcohol ether can form hyper-conjugation with the p-bond of O atom, and the electrons of the C-H bond can delocalize to the orbital of O atom, increasing the electron density around the O atom. O atom with high electron density can attract positively charged Cu^2+^ and form complexes, thereby reducing the catalytic ability of Cu^2+^. Moreover, as shown in Figure 3b, due to the strong electronegativity of the O atom (3.5), the electron pairs between H-O in H_2_O_2_ are heavily biased towards the O atom. The O atom of the alcohol ether attracts the positively charged H atom to form a hydrogen bond, which stabilizes the H_2_O_2_ system.

**Figure 4:**
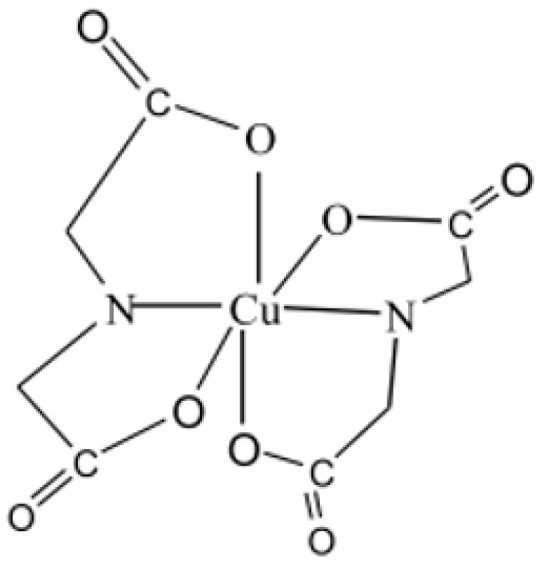
Structure of Cu^2+^-IDA complex.

In addition to adding H_2_O_2_ stabilizer, chelating agents need to be added to the proposed Cu-Mo etchants to chelate the metal ions generated during the etching process. This helps prevent the decline in etching stability and bath life caused by H_2_O_2_ decomposition as Cu^2+^ concentration increases. According to the coordination characteristics of Cu^2+^, a chelating agent that can quickly form stable complexes with Cu^2+^ is needed. The commonly used chelating agents are mainly amino acids, including glycine, alanine, iminodiacetic acid (IDA), and nitrilotriacetic acid. Among them, IDA is more commonly used in TFT-LCD Cu-Mo etchant. Prior study [26] show that the stability of Cu complexes—and, thus, their ability to stabilize H_2_O_2_—varies depending on the chelating agent. Additionally, the solubility of chelating agents and their metal ion complexes differs across the proposed formulations, influencing the overall etching performance. These compounds mainly form complexes with Cu^2+^ through the carboxyl group and N atom [27,28], as shown in Figure 4, stabilizing Cu^2+^ in solution. This prevents Cu^2+^ from participating in oxidation reactions or catalyzing H_2_O_2_ decomposition, thereby extending both the stable etch period and the bath life of the etchant.

### 3.3. Effect of pH on the H_2_O_2_ Stability

To suppress the undercut or residues of Mo or Mo alloys layer, the pH of the etchant can be adjusted appropriately by adding a pH regulator. When etching the Cu/Mo stacks with H_2_O_2_-based etchant, the optimal pH value of the etchant is about 2 to 4 for Cu and about 4 to 7 for Mo. Therefore, maintaining the etchant at the optimal pH for Cu can lead to incomplete Mo etching, causing insoluble Mo oxides and potential residues. Conversely, setting the pH for optimal Mo etching significantly reduces the Cu etch rate. Generally, the pH of Cu-Mo etchant is controlled between 2.5 and 4.5. This balance can be achieved by adding amines or weak alkaline salts in the proposed formulations to control the etch rates of Cu and Mo, avoid Mo residues, and prevent rapid Mo etching and H_2_O_2_ decomposition caused by excessively high pH values. Our research found that when the pH exceeds 5, the stability of H_2_O_2_ decreases, the etch rate of Cu metal lines drops, and the bath life of etchant also shortens.

**Figure 5:**
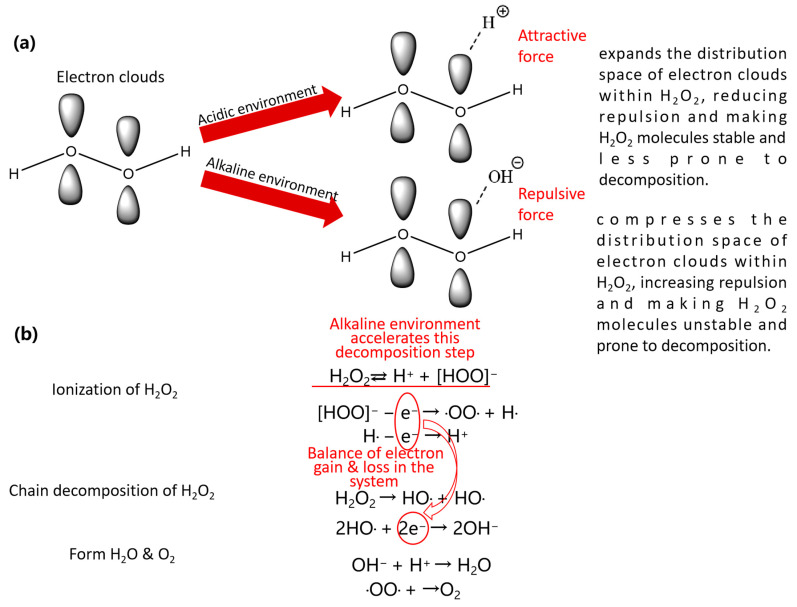
Effect of pH on the stability of H_2_O_2_: (**a**) The influence of H^+^ and OH^−^ on the structure of H_2_O_2_; (**b**) H_2_O_2_ decomposition mode.

The effect of pH on the stability of H_2_O_2_ is shown in Figure 5. From a structural perspective, the electron cloud around the O atom in H_2_O_2_ is dense and spatially constrained, creating strong repulsive force on H atom, making the molecule prone to breaking apart. In acidic environments, H^+^ ions attract electrons, extending the electron cloud range and increasing its distribution space. This reduces the repulsive force between the O and H atoms, stabilizing the H_2_O_2_ molecule and reducing decomposition. In contrast, in alkaline environments, OH^−^ ions repel electrons, compressing the electron cloud range and distribution space. This intensifies the repulsive force between the O and H atoms, making H_2_O_2_ unstable and more susceptible to decomposition. Additionally, although H_2_O_2_ can decompose in both acidic and alkaline environments, alkaline environments accelerate the ionization of H_2_O_2_ → H^+^ + [HOO]^−^. Therefore, as the pH rises, decomposition speeds up, whereas lower pH values enhance H_2_O_2_ stability.

## 4. Results and Discussion

H_2_O_2_-based Cu-Mo etchants, widely used in TFT-LCD manufacturing, typically comprise a multicomponent system including H_2_O_2_, fluoride, peroxide stabilizer, inorganic/organic acid, chelating agent, metal corrosion inhibitor, pH regulators, and other additives. We previously investigated the effects of the type and concentration change in each component in the etchant formulations on the etching performance of Cu/Mo metal stacks. It has been found that addition of an appropriate amount of H_2_O_2_ and fluoride can help improve the etching rates of each metal film, especially in the three-layer structure containing Mo, MoTi or MoNiTi top layer. Adding appropriate inorganic or organic acids improves both etching efficiency and etching quality. The effect of adding different azole corrosion inhibitors on the etching performance of different metal layers varies greatly. Phosphate, as an anti-potential etchant, presents a delicate balance. If its concentration is too low, excessive etching of the Mo or Mo alloy layer can occur, resulting in undercutting; while its concentration is too high, although undercutting is prevented, it is prone to metal tails and residues. Choosing appropriate amine compounds can help stabilize the pH of the etchant. When the amine content is too low, it is prone to Mo residues, while when the content is too high, it would cause a decrease in etching capability.

To comprehensively characterize the developed Cu-Mo etchants (in Table 1), we further studied the influence of etching process conditions on etching performance. We would present the test results about the CD bias variations at different OE, as well as the CD bias variations and taper angle over the bath life. Additionally, we have evaluated critical factors such as the sudden-eruption point, the shelf life, and the tool material compatibility of the developed etchants.

### 4.1. Process Window

In the panel manufacturing process, to ensure complete cleaning of the etched areas and removal of excess materials—thereby ensuring the performance and reliability of the device—the substrate generally undergoes additional etching after EPD, that is OE. If the etchant can etch for various durations within a certain time range while still meeting the required etching performance specifications, this time range is referred to as the “process window” of the etchant. Generally, a wider process window indicates greater etching stability.

**Figure 6:**
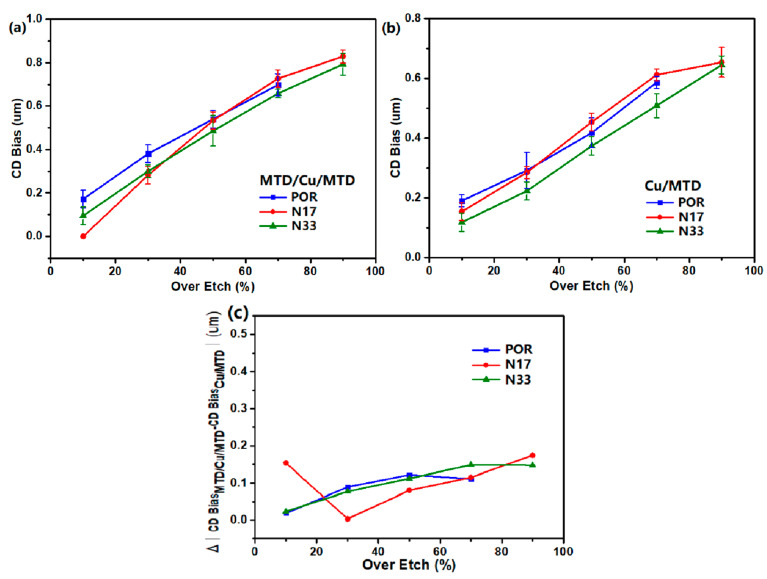
Comparison of etchants N33, N17, and POR at different OE: (**a**) CD bias of MTD/Cu/MTD at different OE; (**b**) CD bias of Cu/MTD at different OE; (**c**) the difference in CD bias between MTD/Cu/MTD and Cu/MTD at different OE. (Cu^2+^ preconditioning levels is 300 ppm.).

We compared the CD bias of MTD/Cu/MTD and Cu/MTD structures after various OE in the developed etchants N17 and N33, as well as the POR etchant used in the panel manufacturing. The results are shown in Figure 6a,b. For OE ranging from 10% to 90%, the CD bias of MTD/Cu/MTD and Cu/MTD after etching in N33 is smaller than that of POR etchant, and the slope of CD bias versus OE is similar to that of POR etchant. Usually, a lower slope of CD bias with respect to over-etch time indicates that CD bias remains relatively stable over a wide etching time range. In addition, from panel manufacturers’ practical experience, the smaller the difference between the CD bias of the MTD/Cu/MTD and the Cu/MTD after etching for the same OE, the better the etching performance of the etchant. As shown in Figure 6c, the CD bias difference between MTD/Cu/MTD and Cu/MTD after etching in N33 is similar to that of the POR etchant. It is found that no undercutting is observed in either MTD/Cu/MTD or Cu/MTD after etching in N33, even for OE from 10% to 90%. Although longer etching time is slightly helpful in improving metal residues, it also increases the CD bias. At present, the POR etchant used in the production line generally achieves a maximum OE of 70%, while the Cu-Mo etchant developed in this study maintains CD bias and CD bias differences across various film structures within process requirements for OE from 10% to 90%. Therefore, compared to the POR etchant, the developed etchant provides an approximately 20% wider process window and demonstrates excellent etching stability.

### 4.2. Cu^2+^ Loading Capacity (Bath Life)

As mentioned above, as the concentration of Cu^2+^ in the etchant gradually increases, the decomposition of H_2_O_2_ accelerates, resulting in a decrease in etching performance and non-uniform etching. The Cu^2+^ loading capacity refers to the maximum concentration of Cu^2+^ that an etchant can sustain under normal etching conditions while maintaining stable performance. This capacity is a key indicator of the etchant’s bath life. In the industry, a Cu^2+^ loading capacity exceeding 6000 ppm is typically required for Cu-Mo etchants to ensure consistent etching quality. This threshold is established based on direct feedback from panel manufacturers, reflecting the practical bath life limit observed in production-scale etching systems. In addition to a high Cu^2+^ loading, minimal variations in CD bias and taper angle throughout the etchant’s bath life are essential to enhance the performance and yield of subsequent processes. For different stack structures and film thicknesses, specific specifications must be met, the CD bias for M2 first wet etch should be between 0.65 and 1.05 μm, while the CD bias for M2 second wet etch should range from 0.5 to 0.9 μm. The taper angle should be between 55° and 70° when the Cu film thickness is less than 5500 Å, and between 60° and 70° when the Cu film thickness is between 5500 and 7000 Å. If the taper angle increases by more than 15° compared to the initial angle or exceeds 70°, yield loss in later processes may rise. Typically, when Cu^2+^ concentration fluctuations cause the taper angle to deviate by more than ±12° or the CD bias by more than ±0.1 μm, the etchant is considered unsuitable and should be replaced.

**Figure 7:**
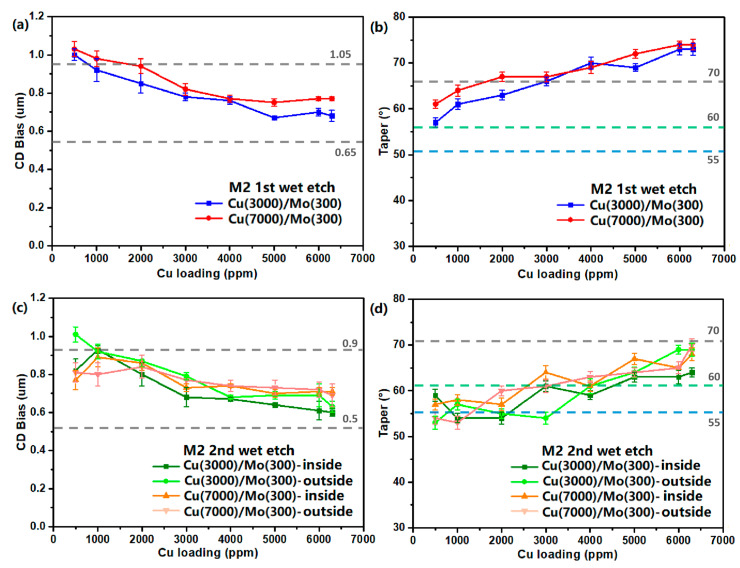
The effect of Cu^2+^ loading in etchant N58-39 on (**a**) CD bias and (**b**) taper angle of M2 1st wet etch samples after etching, etching time: Cu(3000)/Mo(300) 90 s, Cu(7000)/Mo (300) 130 s; and (**c**) CD bias and (**d**) taper angle of the inner and outer sides of the metal lines of M2 2nd wet etch samples after etching, etching time: Cu(3000)/Mo(300) 80 s, Cu(7000)/Mo(300) 110 s.

We have investigated the effects of Cu^2+^ loading in the developed etchant N58-39 on the etching performance of various stack structures with different film thickness. The results, shown in Figure 7, indicate that as the Cu^2+^ loading increases from 500 ppm to 6300 ppm, the CD bias and taper angle, along with their variations, are basically within the specifications, indicating good stability of the etching characteristics. The dashed lines in Figure 7a,c correspond to the upper and lower limits of the CD bias specification, while the dash lines in Figure 7b,d correspond to the upper and lower limits of the taper angle specification, for Cu layer with thickness <5500 Å, the lower limit is 55°, for Cu layer with thickness 5500~7000 Å, the lower limit is 60°. However, for the M2 first wet etch samples, higher Cu^2+^ loading (4000–6300 ppm), resulted in slightly larger taper angles exceeding the specification range. On the contrary, for the M2 second wet etch samples, lower Cu^2+^ loading (500–1000 ppm) led to smaller taper angles below the acceptable range. At present, the Cu^2+^ loading capacity of the POR etchant used in the panel manufacturing is about 5000 ppm, whereas the newly developed etchant achieves approximately 6000 ppm, with a 20% improvement in bath life. Notably, the relationship between Cu^2+^ loading and changes in CD bias or taper angle can be adjusted by modifying the formulation, such as altering the types and concentrations of corrosion inhibitors, inorganic salts, and other additive components.

### 4.3. Shelf Life

Due to the self-decomposition reaction of H_2_O_2_ in the etchant, even with the inclusion of stabilizers and metal chelating agents, the shelf life of high-concentration H_2_O_2_ etchant is usually limited to one month. While a longer shelf life indicates better stability, panel manufacturers generally set the shelf life of Cu-Mo etchants at 15 days. Additionally, they require that upon delivery, the etchant has at least 7 days of remaining shelf life to ensure stable and consistent etching performance, reducing the risk of process defects.

**Figure 8:**
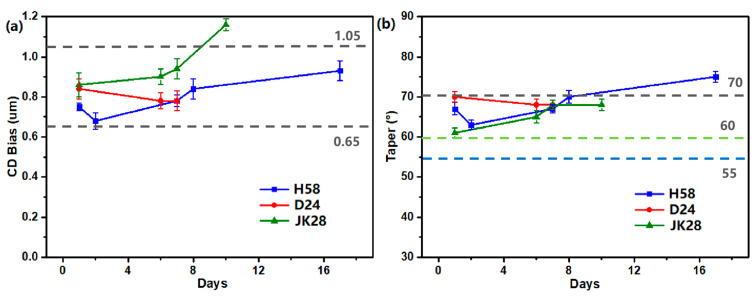
Effect of storage time of etchants D24, H58, and JK28 within their shelf life on (**a**) CD bias and (**b**) taper angle of M2 1st wet etch Cu (5500)/Mo (300) after etching. Cu^2+^ preconditioning levels is 500 ppm. Etching time: 120 s in D24, 130 s in H58, 100 s in JK28.

We have evaluated the effect of storage time of several developed etchants D24, H58, and JK28 on etching characteristics throughout their shelf life. The results in Figure 8 show that the CD bias of M2 first wet etch samples remained within the specification range across all three etchants, even as storage time increased. The dashed lines in Figure 8a and 8b correspond to the upper and lower limits of the CD bias specification and the taper angle specification, respectively. For the Cu layer with thickness <5500 Å, the lower limit of taper specification is 55°, and for the Cu layer with a thickness of 5500~7000 Å, the lower limit is 60°. The CD bias after etching in H58 and JK28 increases with the increase in storage time, while the CD bias after etching in D24 seems to slightly decrease or remain unchanged. The taper angle is also stay within the specification range, except for the etchant H58, where prolonged storage (over 15 days) caused a slightly larger taper angle. Importantly, no undercutting or metal residues are observed after etching in these three etchants within their shelf life. Overall, the etchants demonstrated a shelf life exceeding 15 days with stable performance, meeting the requirements for practical use in Cu/Mo stack etching.

### 4.4. Sudden-Eruption Point

As etch process progresses, the concentration of Cu^2+^ in the H_2_O_2_-based etchant keeps increasing. When it exceeds a critical threshold, H_2_O_2_ rapidly decompose, generating O_2_ and water, releasing significant heat, causing the etchant temperature to rise sharply. When the temperature reaches a certain point, the etchant erupts suddenly, generating high pressure and heat in the container, which may lead to a strong explosion. This phenomenon may pose safety risks in relatively enclosed environments, such as solution tanks in production-line etcher. Therefore, the sudden-eruption point of the etchant, which is equivalent to the highest Cu^2+^ loading of the etchant is explicitly listed in the performance requirements of the etchant. A higher sudden-eruption point indicates better product stability and safety. There are three methods for testing the sudden-eruption point of etchant: (1) Gradually load Cu^2+^ into the etchant at a pace of 1000 ppm per hour at the process temperature until the etchant suddenly erupts, record the curve of temperature change in the etchant over time; (2) load Cu^2+^ up to the maximum bath life capacity, then add an extra 5000 ppm Cu^2+^, measure the temperature change over time; (3) alternatively, a static test can be conducted, that is, load the etchant with the maximum Cu^2+^ concentration for bath life, let it stand for 24 h and monitor the temperature curve.

**Figure 9:**
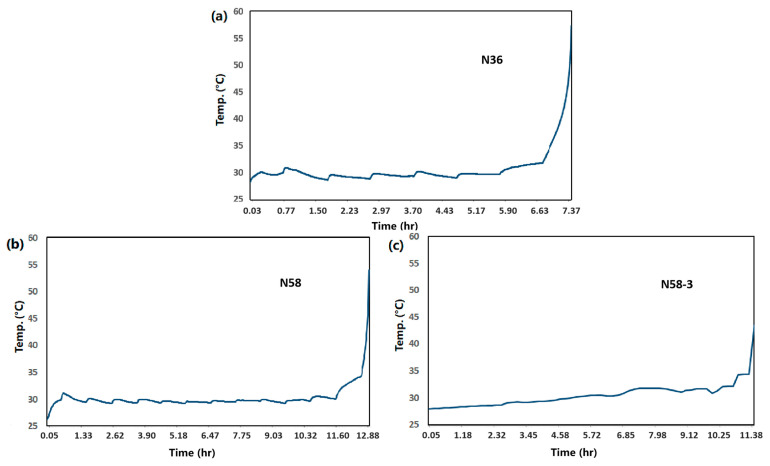
Measurements of sudden-eruption point of etchants: (**a**) N36, (**b**) N58, and (**c**) N58-3—the real-time variation in temperature with Cu^2+^ concentration kept increasing.

We have evaluated the sudden-eruption point of several developed Cu-Mo etchant formulations. The etchants N36, N58, and N58-3 are loaded with 1000 ppm Cu^2+^ per hour until sudden eruption occurred. The real-time change in the etchant temperature is recorded throughout the process. The etchants are stirred and there is no cooling setup for the etchants and environment. The results are shown in Figure 9a–c. The sudden-eruption points of etchants N58 and N58-3 are higher than that of N36, and the phenomenon of sudden eruption occurred only after the Cu^2+^ loading reached 12,000–13,000 ppm and 11,000 ppm, respectively. The experiment results are shown in Table 2. Both N58 and N58-3 demonstrated excellent stability and safety, meeting the industry requirement of a sudden-eruption point greater than bath life + 5000 ppm.

### 4.5. Materials Compatibility

Since the material compatibility of etchant affects the safety, reliability, and durability of the product and equipment, it is a critical aspect of etchant performance. The material compatibility is influenced by various factors, including chemical composition, concentration, volume, temperature, humidity, and contact or exposure time. Among them, temperature plays a significant role, as elevated temperatures can accelerate chemical reactions and alter material properties.

We have evaluated the compatibility of several developed Cu-Mo etchants with various equipment material samples provided by panel manufacturers. There are many methods for testing material compatibility. In this study, various equipment materials have been soaked in the etchant at process temperature of 30 °C for more than 30 days, then taken out, rinsed with deionized water, blow-dried, then oven dried, and finally weighed. The weight changes and the weight change ratios have been measured and calculated. Also, observe the material appearance whether there are obvious changes, such as deformation, dissolution, discoloration or cracking. A weight change ratio of less than 0.2% after soaking for 45 days is considered acceptable. Table 3 shows the results of various material samples after having been soaking in etchants N36 and N58 at 30 °C for 7 weeks (49 days). The weight change ratio for all the materials is less than 0.2%, and almost no change in material appearance, indicating good compatibility of the etchant with equipment materials.

## 5. Conclusions

Through continuously optimizing various formulations of H_2_O_2_ stabilizers, metal chelators, and pH regulators, we developed Cu-Mo etchants, N58 and its variants, for TFT-LCD manufacturing that achieves precise control over etching characteristics of diverse metal/alloy stacks, including CD bias, taper angles, and metal residue elimination. Systematic evaluation of etching time, Cu^2+^ loading, and storage time demonstrated a 20% broader process window compared to the POR etchant, maintaining CD specifications for both MTD/Cu/MTD three-layer and Cu/MTD two-layer even at 10–90% over-etch conditions. The etchants exhibit enhanced Cu^2+^ loading, extending bath life by 20%, while retaining compatibility with industrial equipment materials. Stability testing confirmed a shelf life exceeding 15 days with minimal performance degradation, attributable to synergistic stabilization mechanisms. This study addresses critical bottlenecks in high-precision display manufacturing, with potential applications in next-generation flexible electronics and ultra-high-resolution panels requiring complex multi-material integration.

## Figures and Tables

**Table 1:** Evaluated Etchants Composition Information.

**Component** **Category**	**Component Species**	**Etchant Formulations**							
		**N33**	**N36**	**N58**	**N58-3**	**N58-39**	**D24**	**H58**	**JK28**
Oxidant	H_2_O_2_	●	●	●	●	●	●	●	●
Fluoride	Fluoride	●	●	●	●	●	●	●	●
Organic/Inorganic Acid	iminodiacetic acid	●	●	●	●	●	●		
	sulfamic acid						●		
	malonic acid							●	●
	phosphoric acid							●	
	taurine							●	●
	acetic acid	●	●						
	p-toluenesulfonic acid	●	●						
Azole	aminotetrazole	●					●	●	
	methyl tetrazole		●	●	●	●			
	1,2,4-triazole					●			
	3-amino-1,2,4-triazole								●
Inorganic/Organic Salt	sodium monobasic phosphate	●	●	●	●	●	●		
	sodium dibasic phosphate	●	●						●
	ammonium dibasic phosphate						●		
	potassium acetate	●	●						
	potassium sodium tartrate						●		
Alcohol Ether	triethylene glycol	●	●	●	●	●	●	●	●
Amine	diethanolamine							●	
	triethanolamine								●
Others	surfactant 1								●

**Table 2:** Test results of sudden-eruption point of etchants N36, N58 and N58-3.

Etchant	Number of Experiments	Experiment Duration	Cu Loading at Sudden-Eruption	Single Cu^2+^ Addition per Hour
N36	1st time	6 h 50 min	7000 ppm	1000 ppm/h
	2nd time (repeated test)	7 h 25 min	7000 ppm	1000 ppm/h
N58	1st time	11 h 50 min	12,000 ppm	1000 ppm/h
	2nd time (repeated test)	12 h 58 min	13,000 ppm	1000 ppm/h
N58-3	1st time	11 h 26 min	11,000 ppm	1000 ppm/h

**Table 3:** Compatibility of etchants N36 and N58 with various equipment materials.

Etchant	N36 (@30 °C for 7 Weeks)				N58 (@30 °C for 7 Weeks)			
Type of Materials	Before- Soaking Weight (g)	After- Soaking Weight (g)	ΔWeight (g)	Weight Change Ratio	Before- Soaking Weight (g)	After- Soaking Weight (g)	ΔWeight (g)	Weight Change Ratio
PP	10.8101	10.8124	0.0023	0.0213%	11.4157	11.4186	0.0029	0.0254%
C-PVC	23.4609	23.4817	0.0208	0.0886%	16.4829	16.4965	0.0136	0.0825%
UPE	9.2396	9.2450	0.0054	0.0584%	9.6127	9.6196	0.0069	0.0718%
PTFE	17.8450	17.8479	0.0029	0.0162%	25.5174	25.5239	0.0065	0.0255%

## Data Availability

Data are included within the article.
